# Weight-Bearing MR Imaging as an Option in the Study of Gravitational Effects on the Vocal Tract of Untrained Subjects in Singing Phonation

**DOI:** 10.1371/journal.pone.0112405

**Published:** 2014-11-07

**Authors:** Louisa Traser, Michael Burdumy, Bernhard Richter, Marco Vicari, Matthias Echternach

**Affiliations:** 1 Institute of Musicians' Medicine, University Medical Center, Freiburg, Germany; 2 Department of Oto-Rhino-Laryngology, Head and Neck Surgery, University Medical Center, Freiburg, Germany; 3 Department of Radiology, Medical Physics, University Medical Center, Freiburg, Germany; 4 Fraunhofer MEVIS, Bremen, Germany; 5 Esaote S.p.A., Genoa, Italy; Northwestern University, United States of America

## Abstract

Magnetic Resonance Imaging (MRI) of subjects in a supine position can be used to evaluate the configuration of the vocal tract during phonation. However, studies of *speech* phonation have shown that gravity can affect vocal tract shape and bias measurements. This is one of the reasons that MRI studies of *singing* phonation have used professionally trained singers as subjects, because they are generally considered to be less affected by the supine body position and environmental distractions. A study of untrained singers might not only contribute to the understanding of intuitive singing function and aid the evaluation of potential hazards for vocal health, but also provide insights into the effect of the supine position on singers in general. In the present study, an open configuration 0.25 T MRI system with a rotatable examination bed was used to study the effect of body position in 20 vocally untrained subjects. The subjects were asked to sing sustained tones in both supine and upright body positions on different pitches and in different register conditions. Morphometric measurements were taken from the acquired images of a sagittal slice depicting the vocal tract. The analysis concerning the vocal tract configuration in the two body positions revealed differences in 5 out of 10 measured articulatory parameters. In the upright position the jaw was less protruded, the uvula was elongated, the larynx more tilted and the tongue was positioned more to the front of the mouth than in the supine position. The findings presented are in agreement with several studies on gravitational effects in speech phonation, but contrast with the results of a previous study on professional singers of our group where only minor differences between upright and supine body posture were observed. The present study demonstrates that imaging of the vocal tract using weight-bearing MR imaging is a feasible tool for the study of sustained phonation in singing for vocally untrained subjects.

## Introduction

Over the last several years, Magnetic Resonance Imaging (MRI) has improved the analysis method of dynamic processes of the vocal tract (including the oral cavity, pharynx and epilaryngeal tube), such as swallowing [1], [2], singing voice [3]–[5] and speech [6]–[10].

These studies used clinically available high field MR scanners, because they allow a sagittal slice of the whole vocal tract to be recorded without ionizing radiation. In the scanners the subjects had to perform in a supine position. Since an upright position is the usual position for phonation, the effect of the supine position has been a matter of some research in order to estimate its influence on the vocal tract [11]–[17].

Takemoto et al. described differences between formant frequencies (i.e. spectral peaks in the sound spectrum of the voice) from acoustical analysis of natural speech sounds in the supine position and area functions derived from stable and cine 3D MR images [12]. The authors assigned these differences to changes of the vocal tract geometry caused by the different impact of gravitational forces in the supine position between stable phonation and continuous utterance. In another study on speech phonation by the group, an open type MR system was used (GE 0.5T MRI SIGNA SP/i) and the impact of gravity on the articulatory system was found to be significant [13]. For the three subjects the difference in tongue position was in the range of 3–20 mm and the height of the arytenoid part of the larynx showed a difference in the range of 3–9 mm. The authors also noted that a tongue retraction due to gravity, could be observed.

Stone et al. compared speech production in upright and supine body positions using ultrasound and found that most subjects had small upright-supine differences [14]. However, in keeping with the findings of Kitamura et al. [13], the largest differences (less than 3 mm on average) were found for the position of the posterior tongue, even though compensatory effects of gravity differed across subjects [14]. Using an X-ray microbeam, Tiede et al. found that observed effects on posture were greatest for sustained vowels and smallest for running speech [15].

Recently Steiner et al. supplemented ultrasound tongue imaging with simultaneous electromagnetic articulography in order to examine different vowel and consonant conditions while subjects were exposed to noise in both upright and supine positions [16]. Their preliminary results showed that jaw, lip and tongue tip motion were strongly affected by the supine position and somewhat affected by noise [16], although clear numbers are yet to be published. All of these studies analyzed speech phonation in untrained vocalists. With regards to the singing voice a recent MRI study on professional singers by the present authors showed only few significant effects of gravity on the articulatory system during singing voice production [17] in supine and upright positions. Only the vertical larynx position was found to be significantly higher and the jaw more protruded in the supine position. These results fit with the general assumption that professional voice users are less affected by the situations with a clinical high field MRI system, i.e. the supine body position, noisy environment and confined space. Consequently, in most MRI studies on singing phonation, only professional trained voices were included [18].

As can be seen from these studies, imaging modalities other than MRI have been used to compare the vocal tract geometry in upright and supine position, as they do not have the limitations of an unmovable large magnet. Among these, ultrasound and optoelectronic position measurements provide only a partial view of the vocal tract and have to focus on specific articulators, whereas X-ray exposes the subject to harmful radiation. Weight-bearing MR imaging with a rotatable, open configurated MR system allows measurements in the upright and supine position, while providing a view of the whole vocal tract without ionizing radiation. Since such systems are less noisy than high field systems, the use of hearing protection is neither necessary nor recommended by the scanner manufacturers.

In summary, the findings of previous studies on speech phonation suggest that the supine position causes changes to the vocal tract shape of speakers, whereas the study of professional singers showed fewer differences caused by this position. This is a discrepancy which could be related to the type of phonation (speech or singing) or the status of training (untrained or trained voice production), as none of the speech subjects were professionally trained speakers. Consequently, the study of singing phonation in vocally untrained subjects could help to answer this question, by comparing these results with existing studies of speakers and professional singers. Secondarily, such a study will also increase knowledge of intuitive singing functions, i.e. singing behaviour that was not specifically taught to the person, e.g. raising of the larynx with pitch.

The present study aims to analyze the feasibility of an open-configuration, weight-bearing 0.25 T MRI system with a rotatable examination bed in order to compare untrained subjects' singing voice production in upright and supine positions. As a hypothesis, it is assumed that untrained subjects will show significant changes in the articulators in response to the different gravitational forces while singing in supine and upright positions.

## Material and Methods

Twenty healthy subjects (all male, age 24–46 years, mean 32 years) participated in the study. None of the subjects had ever received singing lessons. At the time of the recording none of the participants complained of any vocal symptoms, such as hoarseness or vocal fatigue.

**Figure 1:**
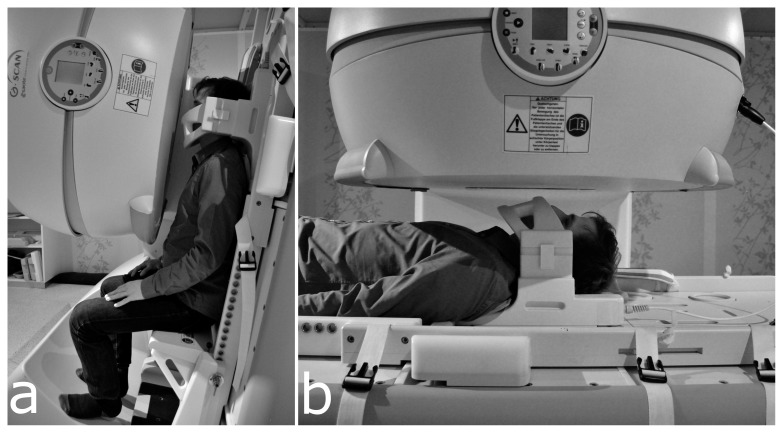
Two Positions of a Participant in the G-Scan. The upright (a) and supine (b) positions of a subject in the G-Scan during measurements are depicted. The head is located in the isocentre of the magnet, in a dedicated 2-channel phased array neck coil. The upper half of the system, containing parts of the permanent magnet and gradient system, is located in front of the head. The individual in this figure gave written informed consent (as outlined in PLOS consent form) to publish these case details.

All participants were examined with an Esaote G-Scan MRI system [19] (Esaote S.p.A., Genoa, Italy) which features a 0.25 T permanent tilting magnet that can be rotated from 0° to 90° (see figure 1). The gradients support a maximum intensity of 20 mT/m with a slew rate of 25 mT/m/ms. A dedicated 2-channel phased array neck coil was used to acquire one sagittal slice through the midsection of the subject's head. The following sequence was used: Balanced steady state free precession sequence, TR = 10 ms, TE = 5 ms, Flip Angle = 80°, FOV 300×300 mm, Matrix = 212×212, Pixel Size 1.42 mm, Slice Thickness  = 8 mm, with two RF phase cycles. Variable-density k-space under-sampling with constrained image reconstruction provided an acceleration factor of 1.5, leading to an acquisition time of 3 seconds per frame. In-plane gradient non-linearity distortion correction was applied.

### Ethics Statement

The Medical Ethics Committee of University of Freiburg, Germany, approved this study (Nr. 206/09). Subjects gave written informed consent prior to participation. The examiner conducting the consent process provided a description of the protocol and outlined the potential risks and benefits of participation.

### Task

In this study we defined “singing” as a constant phonation on a given pitch, loudness and register, without claims to musical quality. In accordance with previous studies of professional tenors [17] the participants were asked to sing an ascending diatonic major scale from C4 (262 Hz) to A4 (440 Hz) on the vowel/a/at a comfortable volume. The subjects were instructed to sing the first three tones (C4 to E4, later referred to as ‘higher modal register’) in their modal register and the last three tones (F4 to A4) in their falsetto register, since it was expected that untrained subjects would not reach the higher notes in modal register. Additionally, the subjects were asked to sing the note C3 (130 Hz, later referred to as ‘lower modal register’). The task was first performed in an upright sitting position at an angle of 80° (see figure 1a) and then the subject was rotated into a supine position (0°, see figure 1b) and the task repeated.

The complete examination was undertaken in a single recording session, and the subjects stayed on the patient bed inside the scanner while the magnet was rotated. The subjects were asked to move as little as possible during and after the rotation. The subject's face was at the edge of the measurable field of view of the scanner due to the reduced magnet homogeneity region (25×25 cm) compared to the high field MR scanners and the coil layout that was optimized for spine imaging. Thus, even small movements of the head would have been noticed by the examiner. In the case of the G-Scan, the examination room was separated from the control room by a thin perforated metal wall that acts as a faraday cage. It was therefore possible for the examiner to maintain constant visual and auditory contact with the subject.

Since all the participants were vocally untrained, the tasks to be performed and the terminology used in the protocol were explained in detail before the session. The pitch was checked with a pitch generator and sung by one of the examiners (who were both trained singers) to the participants immediately before each single tone measurement, as the subjects found it much easier to imitate pitches given by a real voice. The subjects were then asked to sing the note a few times, until the two examiners were certain that the correct pitch and register was reached. To minimize the influence of masking by the scanner noise, the subjects first started to sing and the scanner sequence was then started after two seconds. Due to the low scanner noise the examiners were able to hear the performance during the scan and were able to check the requested task concerning the pitch and register.

Images were accepted for analysis only if the examiners agreed that the vocalizations were performed as instructed. The vowel/a/was chosen to avoid possible articulatory effects which can be expected when the pitch exceeds the normal value of the first resonance frequency of the vocal tract, the first formant [3], [5].

### Anatomical Measurements and statistical evaluation

**Figure 2:**
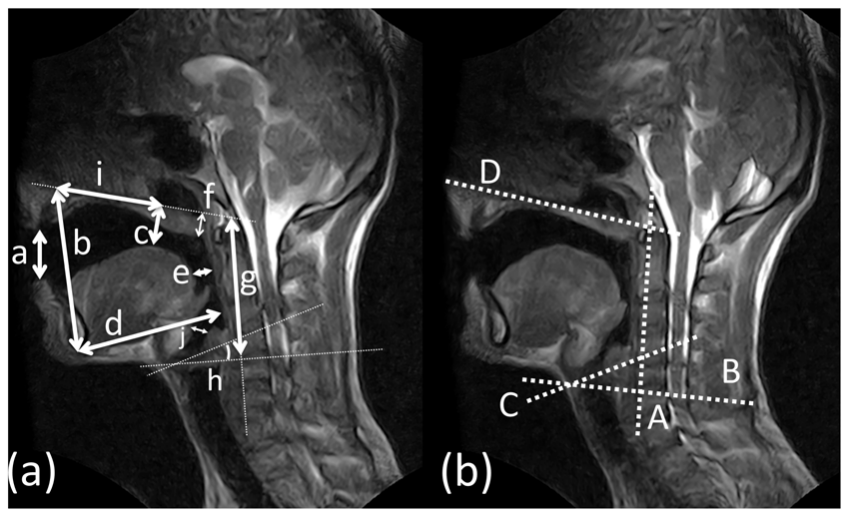
Vocal tract measurements. MR images of the vocal tract in sagittal orientation of a subject in the supine (a) and upright (b) position, using a balanced Steady State Free Precession Sequence. The subject held the note G4 on vowel/a/. Anatomical distance measurements are displayed with white lines and annotations. The dotted auxiliary lines are marked (A-D); measured distances are displayed by white lines with arrows and marked by small letters: a  =  lip opening, b  =  jaw opening, c  =  distance between the tongue and the jaw, d  =  jaw protrusion, e  =  oropharynx width, f  =  uvula elevation, g  =  larynx position, h  =  angle of larynx tilt, i  =  position of the highest point of the tongue (from the front) and j =  epilaryngeal tube. A more detailed explanation of the parameters measured can be found in the methods section.

Several landmarks were measured for all images by an anatomical expert, as previously described [4]. Figure 2 gives an overview of the chosen measures. In order to calculate the distances, four auxiliary lines were necessary. Line A connects the cranial-most part of the dens axis (second cervical vertebra, C2, of the spine) and the caudo-anterior edge of the sixth vertebra (C6). Line B runs through the anterior commissure perpendicular to auxiliary line A. Line C runs through the anterior commissure and follows the angle of the vocal folds. Line D defines the upper contour of the hard palate and runs through the dens axis. The use of these auxiliary lines allowed greater comparability of measurements of different subjects with different head tiltings.

The following distances were measured:

(a) lip opening, defined as the vertical distance between the lowest point of the upper lip and the highest point of the lower lip,

(b) jaw opening, defined as the distance between the spina of the upper jaw and the lower front edge of the mandible,

(c) distance between the highest point of the tongue and the jaw, defined as the shortest distance between auxiliary line D and all points on the tongue's surface,

(d) jaw protrusion, defined as the shortest distance between the lower front edge of the mandible and the mucosal cover of the spine,

(e) oropharynx width, defined as the shortest dorsal-ventral distance between the posterior contour of the tongue, and the mucosal cover of the spine,

(f) uvula elevation, defined as the distance between line D and the lowermost part of the uvula contour,

(g) larynx position, defined as the distance from the cranial-most part of the dens axis to the point where line A crosses line B,

(h) laryngeal tilt, defined as the angle between line A and line C,

(i) Distance to the highest point of the tongue seen from the front, defined as the distance between the most anterior part of auxiliary line D and the intersection point of line D and the line defined by distance (c),

(j) epilaryngeal tube, defined as the shortest distance between the arytenoid cartilage and the epiglottis.

Even though in most cases more than one image could be acquired for each sung pitch, only one image from the middle part of the phonation was used for the anatomical measurements.

For statistical analysis the anatomical measurements of the different articulatory parameters were grouped into lower modal (C3), higher modal (C4-E4) and falsetto register (F4-A4), which will be referred to in the following as ‘pitch groups’. Only subjects who succeeded in the whole requested task were taken into account for statistical evaluation (n = 15).

A one-way between subject analysis of variance (ANOVA) was performed in order to investigate the effect of pitch group on the vocal tract measurements. Significant results were analyzed using a post-hoc t-test (Tukey HSD).

A repeated measure ANOVA with the factors of body position and pitch group was performed in order to evaluate the possible impact of the body position within pitch group on the measured parameters.

The individual differences between the upright and supine body positions were evaluated for all pitches and separately for each pitch group using a paired student t-test. Formant frequencies taken from the noise distraction experiment (see Evaluation of Noise Distraction) with and without masking were compared using a paired student t-test. Articulatory data for the three subjects tested and the 12 subjects who were not tested in the noise distraction experiment were compared using a test for intersubjective effects. All statistical tests were performed with IBM SPSS Statistics (Version 22.0. Armonk, NY: IBM Corp) with the level of significance set to 0.05. As multiple measurements with different variables were performed for the comparison of upright and supine position, the level of significance was reduced to p = 0.02 to avoid a multiple testing problem.

### Evaluation of Noise Distraction

It is possible that the noise of the scanner, in addition to body position, has an influence on vocal tract configuration, articulator position and formant frequencies due to a masking effect.

The noise level of the empty scanner during the test sequence was measured with a calibrated microphone (Prepolarized Free-field 1/2" Microphone Type 4189, Brüel&Kjær, Nærum, Denmark) positioned in the isocentre of the magnet, where the head of the subject would be. The signal voltage of the microphone transducer was then recorded via an oscilloscope with the patient bed in vertical (80°) and horizontal (0°) positions and processed with Matlab (2012b, The MathWorks, Inc., Natick, USA) to calculate absolute A-weighted sound pressure levels (SPL).

In order to evaluate any further possible distraction to subjects caused by the scanner noise, three subjects chosen at random were tested additionally in a sound-proof room. The subjects were asked to perform the same task as in the MR measurement in both upright and supine body positions, with and without auditory masking by broad-band noise of 85 dB(A), the (rounded) SPL measured at the scanner. As was the case in the MR system, the participants first started to sing and the masking started after two seconds. Electroglottographic and audio signals, air flow and air pressure were simultaneously recorded using a Laryngograph MicroProcessor (sampling rate 16 kHz, Laryngograph, London, UK) combined with the MS 110 (pressure transducer PT-70, flow transducer PT-2, MA- 1L Rothenberg mask, Glottal Enterprises, NY, USA).

Since vocal formants are strictly dependent on the shape of the vocal tract, any change in vocal tract shape caused by auditory masking would be reflected in an alteration of the measured formants. The flow signal was therefore analysed through inverse filtering using DeCap software (Svante Granquist, KTH, Stockholm, Sweden), as described previously [20].

## Results

For all the subjects the acquisition time of three seconds was short enough to allow them to phonate a stable tone in the upright, as well as in the supine body position during the MR measurement. The required task was fully accomplished by 15 of the 20 untrained subjects, and thus the data of 5 subjects could not be considered in the statistical analysis because the requirements for either register or pitch or both were not met.

**Figure 3:**
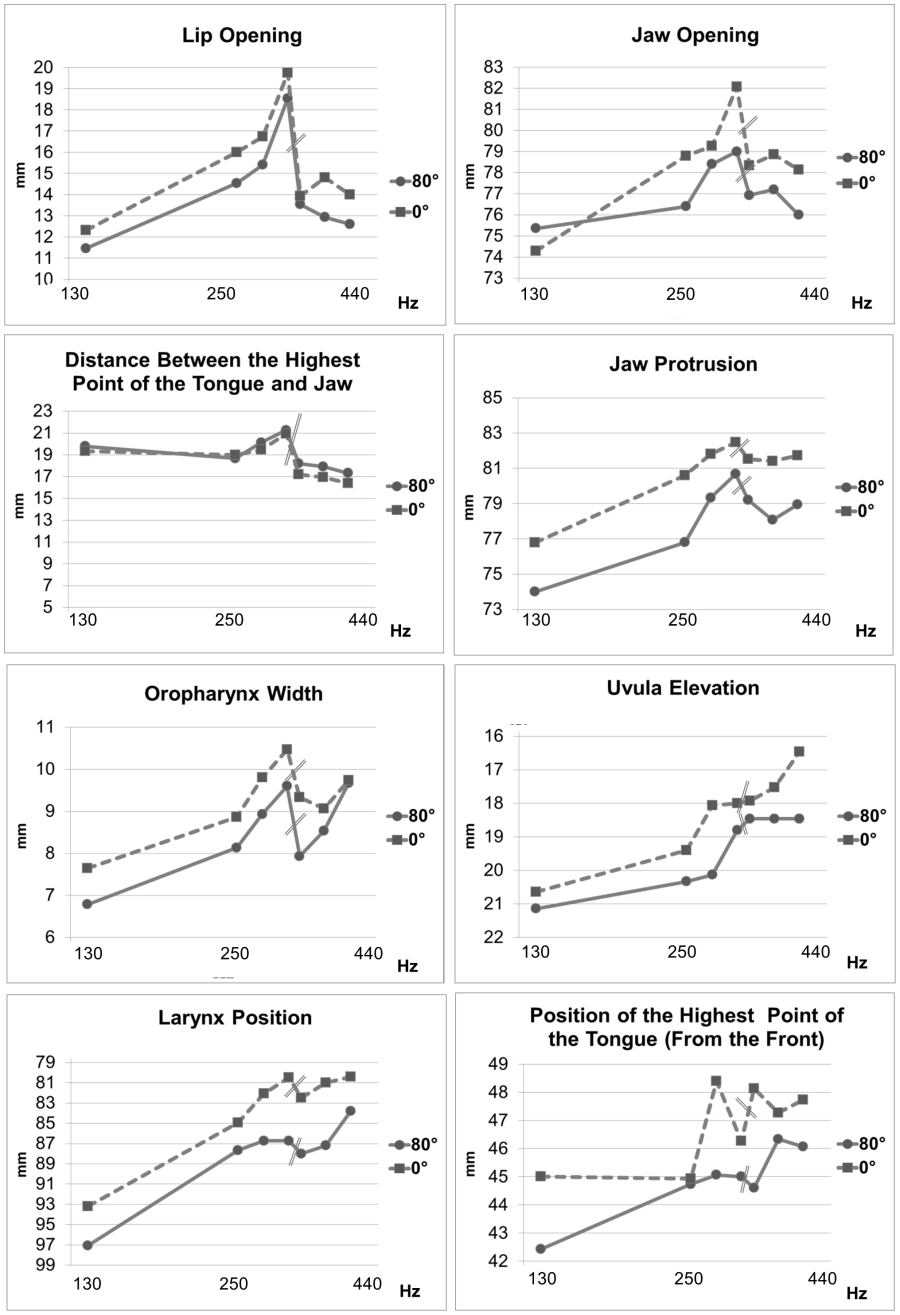
Articulatory parameters and pitch. Mean values of all articulatory parameters measured (see Figure 2) for the 15 subjects completing the task. Data points are ordered by fundamental frequency and are plotted on a logarithmic scale. The x-locations of the data points correspond to pitches C3, C4-A4. A double bar indicates the register change from higher modal to falsetto register. Dashed lines refer to the supine position (0°) and solid lines refer to the upright body position (80°).

Figure 3 displays the mean values of the articulatory parameters for each pitch for the 15 participants who completed the task in both body positions. The mean values are connected by either a solid (upright, 80°) or dashed line (supine, 0°) so that the difference of the measured parameters across the conditions can be observed in a graph according to the ascending scale.

### Vocal Tract Modifications Between Pitch Groups - Higher, Lower Modal and Falsetto Register

**Figure 4:**
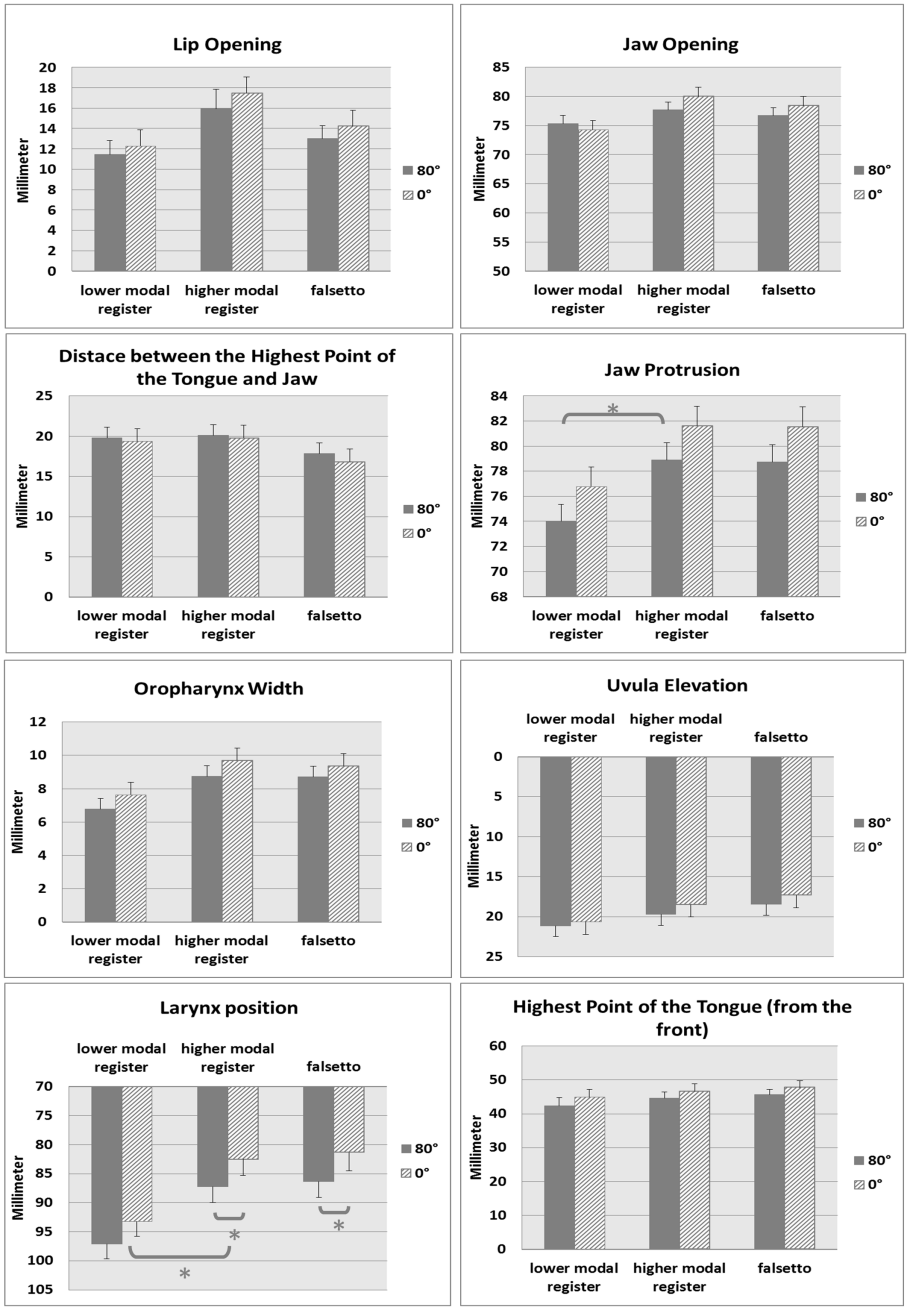
Mean values and standard error for evaluated pitch groups. Separate diagrams for all measured parameters, depicting the mean values and standard errors of all subjects for the evaluated pitch groups lower modal register, higher modal register and falsetto, ordered by fundamental frequency. Only data from the 15 subjects completing the tasks was included. The dashed bars refer to the supine position (0°) and solid bars refer to the upright body position (80°). Significant differences are indicated by “*”.

Figure 4 displays the vocal tract shape modifications between the different pitch groups (higher modal, lower modal and falsetto register). The subjects showed a significant modification of vocal tract shape between different pitch groups for the parameters for jaw protrusion and larynx position in the upright and supine positions. The mean values and standard errors of all the articulatory parameters for the evaluated pitch groups for the 15 subjects who completed the tasks are illustrated in the figure. Comparing the data for the lower and higher modal register, the jaw was more protruded (upright: p = 0.04; supine: p = 0.04) and the larynx elevated (upright: p = 0.04 and supine: p = 0.03) in the higher modal register. No significant shape modifications could be observed when the subjects sang the tasks from higher modal to falsetto register.

### Vocal Tract Modifications Between the Upright and Supine Body Positions

Table 1 presents an overview of the p-values of the paired student t-test regarding the differences in the morphometric measurements between the upright and supine body positions. For all pitches, jaw protrusion, uvula elevation, larynx position, angle of larynx tilt and highest point of the tongue were significantly different. In the upright position the larynx was positioned significantly lower and was more tilted, the jaw less protruded, the uvula less elevated, and the highest point of the tongue positioned more to the front than in the supine position. Within pitch groups for the higher modal register, larynx tilt and larynx position differed significantly. In falsetto register, larynx position alone was significantly different. None of the parameters differed significantly in the lower modal register.

**Table 1:** P values of paired student T-test concerning the differences of the articulatory parameters between the supine (0°) and the upright (80°) body positions in all pitches and pitch groups.

	All pitches	C3	C4-E4	F4-A4
Lip opening	0.024	0.447	0.072	0.178
Jaw opening	0.127	0.463	0.058	0.049
Distance between the highest point of the tongue and the jaw	0.170	0.670	0.536	0.158
Jaw protrusion	**<0.001**	0.025	0.057	0.029
Oropharynx width	0.021	0.207	0.072	0.344
Uvula elevation	**0.009**	0.530	0.045	0.041
Larynx position	**<0.001**	0.025	**0.007**	**0.001**
Angle of lanrynx tilt	**0.011**	0.222	**0.006**	0.444
Highest point of the tongue seen from a front view	**0.005**	0.071	0.215	0.073
Epilaryngeal tube	0.930	0.664	0.480	0.820

Significant p values (p<0.02) are in bold print.

### Noise Distraction Measurements

The calculated A-weighted continuous SPL near the ear of the subjects during a running sequence was 83.8 dB(A) with an upright and 82.1 dB(A) with a horizontal patient bed. During the experiment in the sound-proof room no significant influence of noise was found on the frequency of the lower two formants (1^st^ formant: p = 0.32 in supine and 0.43 in upright position and 2^nd^ formant: p = 0.11 in supine and 0.36 in upright position). For all the three subjects a high and significant correlation was found between the formants with and without the noise distraction in both body positions (1^st^ formant: p = 0.003 with r = 0.78 in the supine and p = 0.04 with r = 0.59 in the upright position and 2^nd^ formant: p = 0.01 with r = 0.71 in the supine and p = 0.007 with r = 0.73 in the upright position). Articulatory data were also compared between the three subjects tested and the 12 subjects who were not tested in the noise distraction measurements. Here we found comparable articulatory changes with regard to the body positions and tasks.

## Discussion

This study demonstrates the feasibility of using an open-configuration, tilting, 0.25 T MRI system for vocal tract imaging in untrained subjects and presents quantitative data of vocal tract modifications in the upright and the supine body positions. In general, it was found that similar to speech phonation body position does have an effect on the vocal tract during singing in vocally untrained subjects.

In comparison to previous studies which examined gravitational effects on human voice production, the present research employed an MR system which allowed measurements in both the upright and supine positions within one session. The subject did not have to exit the system during the rotation of the magnet. This facilitated the comparison of the images obtained, as a different positioning of the patient within the coil could be ruled out. Additionally, the subjects were asked to move as little as possible during and after the rotation and to keep their head against the patient bed at all times. The available neck coil that was employed in this study provided a complete visualization of the vocal tract in a closely defined area. Any change in head position was noticed immediately by the examiners. Changes of the spinal curvature were only minimal between upright and supine positions, as can be seen in Figure 2. This positioning of the subject's head could be an important factor when the vocal tract is compared in both body positions, as the spinal curvature can contribute to the voluntary use of vocal function [21].

The open environment of the G-Scan provides a more comfortable environment for subjects than the confined enclosures of high field scanners. In addition the more direct contact with the examiner simplifies task-related measurements. The continuous SPL measured near-the-ear during the running sequence inside the MR system was 85 dB(A) whereas a sample second measurement with a real time FLASH sequence [4] in a high field system registered 99 dB(A), a noise level frequently reached in night clubs [22]. Thus, the measurement in the G-Scan was less noisy than the compared sequence in the high field scanner. In general, subjects in the G-Scan require no hearing protection, since all sequences are considered to be quiet enough not to harm the subject's hearing.

Auditory masking by noise can have an influence on the articulators and bias the measurements in both upright and the supine positions. However, all the subjects started the phonation in a quiet environment, before the sequence was initiated. The sequence and related noise started when the subjects were already singing. The subject's performance could always be heard over the scanner noise by the examiners and no alteration of pitch or volume was audible. To gain an estimate of error, an additional noise distraction experiment was performed and formant frequencies with or without masking of 85 dB(A) were calculated and found to be comparable. Since formants are strictly dependent on vocal tract shape, it could be concluded that, at least for these three subjects, the vocal tract was not significantly influenced by the noise. However, not all of the subjects were tested and even small differences might have been significant if all subjects had been included.

During the experiment inside the MR scanner the subjects performed in a familiar (upright) and unfamiliar (supine) singing position. The smallest measured parameter was 3 mm (the oropharynx width), while most of the measured parameters were much greater (45 mm on average). As such, the chosen spatial resolution of 1.42 mm per pixel for the acquired images was sufficient for the anatomical distance measurements. All the untrained subjects were also able to hold a note for three seconds, which is the time that was required to acquire a single image. Due to the small magnetic field of the G-Scan and the low signal to noise ratio, a further increase of the spatial and temporal resolution would only be possible with improved imaging hardware.

When different pitches were compared, major changes in vocal tract configuration between the subjects' lower modal and the higher modal register were found. In contrast, singing from higher modal to falsetto register showed no significant alteration in vocal tract shape. These data are comparable to professional opera tenors singing the same task [4]. Previous studies of vocal tract length and vertical laryngeal position have also shown an elevation of the larynx with pitch in untrained subjects. The larynx elevation is considered to lead to an increase in vertical tension with an increase in vocal fold stiffness and thus in fundamental frequency [21], [23]. In contrast to professional singers, untrained singers did not raise their larynx significantly between the pitches C4 in modal to F4 in falsetto register. It might therefore be speculated that the untrained subjects had already reached the highest anatomically possible larynx position singing C4, suggesting that no further elevation was possible for the following tones.

The hypothesis that significant modifications of the vocal tract shape might occur due to gravitational effects is supported in 5 out of 10 parameters. It therefore seems that there is indeed some influence of gravity on untrained subjects in both singing and speech. The present study found a significant difference in tongue position for the lower modal and falsetto register alone, whilst, in contrast, the higher modal register showed no difference in tongue position between the upright and supine body position. It can therefore be assumed that the muscle tension in the tongue, due to the higher effort involved, is probably greater for higher pitches in untrained voices and higher in modal register compared to falsetto register. Thus, the tongue might be more vulnerable to gravitational effects for lower modal singing or singing in falsetto register as well as in speech phonation. The position seems to be more stable in higher pitch singing phonation in modal register. This assumption is also supported by the study of Stone et al., who reported an average difference of 3 mm in the posterior tongue position in speech [24]. Kitamura et al. investigated the retraction of the tongue in the supine position in vowel articulation [13]. They found that the highest point of the tongue was positioned backwards in the supine position. The same study reported that the vertical larynx displacement between supine and upright positions was between 3 and 9 mm, and higher in the supine position [13]. Their results concerning the larynx position are also in good agreement with the data presented in this study, which implies that vocal tract length depends on body posture and may be an expression of the reduced tracheal pull in the supine position.

In contrast to speech phonation in untrained subjects, professional singers showed only minor differences with regards to vocal tract configuration between the upright and supine positions [17]. For both untrained singers and professional tenors the jaw was less protruded and the pharynx narrower in the upright position. This finding was unexpected. We speculate that the subjects intuitively overcompensated to counteract the gravitational downward force. Alternatively, differences of the head inflection between the upright and supine body positions could have reduced the free space for the jaw. An altered position would then force the subjects to protrude the jaw to be able to open the mouth wider for higher pitches and, secondarily, widen the pharynx. However, the differences in the head inflection were small, as seen in Figure 2.

In the present study, the untrained subjects presented an elongated uvula, a more tilted larynx and the highest point of the tongue was positioned more to the front of the mouth in the upright position. This change in tongue position is in agreement with other studies of speech phonation and could be directly related to the force of gravity, as is similarly found for uvula length. The tracheal pull, which is reduced in the supine position, is known to be an antagonist of the cricothyroid muscle [25] and could lead to increased activity and tilting of the larynx in the upright position. The differing articulatory changes in professional and untrained singers suggest that the special vocal training of professional singers might help to counteract gravitational effects on the vocal tract.

Nevertheless, in the case of untrained singers, the measured parameters were significantly different in the upright and supine positions, and one can observe that when a parameter changed depending on pitch in one position (e.g., enhanced jaw protrusion between the lower and higher modal registers) it would also change about the same amount in the same direction in the other position (see Figure 3). Hence, the qualitative behavior of the articulators was the same, albeit with an offset caused by gravity (which can be visualized when the graphs in figure 3 show a parallel trajectory). Our results suggest that quantitative studies of untrained subjects in the supine position in high field scanners are generally possible, but gravitational differences have to be quantified and accounted for.

In conclusion, imaging of the vocal tract using weight-bearing MR imaging is a feasible method for quantifying modifications of the vocal tract configuration in vocally untrained subjects for sustained phonation, with the advantages that subjects can be analyzed in both a natural upright and a supine position and that subjects are exposed to less noise than in high field scanners.
